# Comparison of Root Filling Quality of Two Types of Single Cone-Based Canal Filling Methods in Complex Root Canal Anatomies: The Ultrasonic Vibration and Thermo-Hydrodynamic Obturation versus Single-Cone Technique

**DOI:** 10.3390/ma14206036

**Published:** 2021-10-13

**Authors:** Yong-Sik Cho, Youngjun Kwak, Su-Jung Shin

**Affiliations:** 1 Department of Conservative Dentistry, College of Dentistry, Yonsei University, Seoul 03722, Korea; endocho@naver.com; 2 Private Practice—Yonsei Doctors’ Dental Clinic, 201, 28 Daesan-ro, Ilsandong-gu, Goyang-si 10359, Korea; 3 Private Practice—Yonsei Nature Dental Clinic, 2F, 281 Yangnyeong-ro, Dongjak-gu, Seoul 06919, Korea; ys_nature@naver.com; 4 Gangnam Severance Hospital, Department of Conservative Dentistry and Oral Science Research Center, Yonsei University College of Dentistry, 211 Eonjuro, Gangnam-gu, Seoul 06273, Korea

**Keywords:** calcium silicate-based sealer, canal filling, micro-computed tomography, single-cone technique, VibraTHO

## Abstract

This study aimed to assess the effectiveness of ultrasonic vibration and thermo-hydrodynamic obturation (VibraTHO) using two types of root canal sealers, in comparison to the single-cone (SC) technique and a calcium silicate-based root canal sealer in complex root canal anatomies. Thirty single-rooted human maxillary premolars with two canals that had a complex root canal anatomy of transverse anastomoses or ramifications were prepared and assigned to the following three experimental groups, according to the filling method: SE group, SC technique with Endoseal TCS; VE group, VibraTHO with Endoseal TCS; and VG group, VibraTHO with GuttaFlow 2. Each tooth was scanned using micro-computed tomography, and the volume percentages of the filling material were calculated. The analysis of variance was used to analyze the statistical differences between the three groups (*p* < 0.05). The mean volume of the filling material was higher in the VG and VE groups than that in the SE group (*p* < 0.05) along the apical to middle-to-coronal thirds, and significant differences were observed between each root canal area (*p* < 0.05), with the only exception being at the apical thirds between the VE and SE groups. The VibraTHO technique using GuttaFlow 2 can be a more effective root canal filling method for anatomically complex root canal systems than the SC technique with Endoseal TCS. On the other hand, the VibraTHO technique using Endoseal TCS has a limited effect on improving the quality of the root filling at the apical portion of anatomically complex root canal systems, compared to the SC technique with Endoseal TCS.

## 1. Introduction

Three-dimensional obturation of the root canal system constitutes one of the most important steps in root canal treatment and ensures predictable long-term results [1,2]. Several root canal filling techniques that utilize gutta-percha (GP) and sealers exist; these range from the most widely used cold lateral condensation technique to various modified versions of the warm vertical condensation technique [1,3]. While these techniques focus mainly on filling and packing the GP into the root canal system as much as possible [4], studies have shown that anatomically complex root canal systems can neither be cleaned easily nor filled completely [4,5,6].

Advancements in rotary nickel–titanium instruments have promoted the concept of a single cone (SC) root canal filling because of the availability of master GP cones that correspond to the taper of the rotary file [7]. This concept has become increasingly popular, owing to its simplicity and short procedural time [8]. Recently, the SC technique has been gaining popularity among clinicians, accompanied by an increase in the use of calcium silicate-based root canal sealers [8,9,10,11]. Endoseal TCS (Maruchi, Wonju, Korea) is one of the latest, premixed, ready-to-use calcium silicate paste endodontic sealers, which is supplied in syringes. It is a white-colored version of the Endoseal mineral trioxide aggregate (Maruchi, Wonju, Korea), which has demonstrated acceptable biocompatibility and satisfactory physical properties [12]. The GuttaFlow system (Coltene/Whaledent GmbH + Co. KG, Langenau, Germany) was first introduced in 2004 (before calcium silicate-based sealers); it consists of a cold, flowable, and self-curing obturation material that is intended for use with the SC technique. GuttaFlow 2 is a modification of the RoekoSeal Automix (Roeko Dental Products, Langenau, Germany) and GuttaFlow systems. It is a silicone-based root canal sealer that combines sealer and GP in powder form. While earlier studies have demonstrated the long-term, consistent sealing abilities of GuttaFlow 2 [13,14], the most recent studies have verified that it has excellent thixotropic properties that allow it to flow into very narrow and irregular root canals in response to root filling compaction pressure [15].

In 2021, a novel root canal filling technique, known as ultrasonic vibration and thermo-hydrodynamic obturation (VibraTHO), was introduced [16]. The VibraTHO technique incorporates indirect ultrasonic sealer activation and short-range warm vertical compaction of a single GP cone. This technique is designed to use hydraulic pressure to induce hydrodynamic movement of the root canal sealer within the canal using ultrasonic energy, instead of merely packing the GP itself, as the principal component of the root canal filling. This technique is almost as fast and user-friendly as the conventional SC technique.

The aim of the present ex vivo study was to investigate the effectiveness of the VibraTHO technique in complex root canal anatomies by comparing the root filling quality of the technique using two types of root canal sealers with that of the SC technique using a calcium silicate-based root canal sealer. Micro-computed tomography (micro-CT) scanning was used to evaluate the volumetric percentage of the root canal fillings [8,15,17,18]. Random light microscopic evaluation was used to verify and validate the method.

## 2. Materials and Methods

### 2.1. Sample Size Calculation

G*Power 3.1.9.6 (Universitat Kiel, Kiel, Germany) was used for a sample calculation. Based on the results of our previous pilot test with a sample size of 3 in each group, the estimated sample size in each group was 10 (effect size: 1.804, alpha error: 0.05, power: 95%).

### 2.2. Selection and Preparation of Specimens

A total of 30 single-rooted human maxillary premolars with mature apices and a long, ovoid, or ribbon-shaped cross-sectional root anatomy were collected after obtaining informed consent from the patients under a protocol approved by the Institutional Review Board of Gangnam Severance Hospital (3-2019-0066). Each tooth was selected using radiographic analysis to ensure that it had two canals with a complex root canal anatomy of transverse anastomoses or ramifications. Root canal anatomies were further verified during the preparation of the specimens.

The access cavity was prepared and the working length was established as 0.5 mm short of the apical foramen after coronal flaring of the root canal with a HyFlex CM #25/.08 (Coltene/Whaledent AG, Altstätten, Switzerland) nickel–titanium rotary file. All root canals were prepared using the ProTaper Next system (Dentsply Sirona, Ballaigues, Switzerland) from X1 to X3. Instrumentation was performed under 5.25% NaOCl solution irrigation. The smear layer was removed with a 2 min application of 17% ethylenediaminetetraacetic acid (MD-Cleanser, META BIOMED Co. Ltd., Cheongju-si, Korea) after the completion of instrumentation, which was followed by a final rinsing with 10 mL of 5.25% NaOCl. The contact time for 5.25% NaOCl during the final rinse was 20 min. The irrigant was changed periodically at 5 min intervals, accompanied by recapitulation and patency filing. Apical patency was maintained with a size #15 K-file throughout the procedure. Subsequently, the teeth were divided into three groups (n = 10) for the root canal filling after drying the root canals with paper points (Table 1).

**Figure 1:**
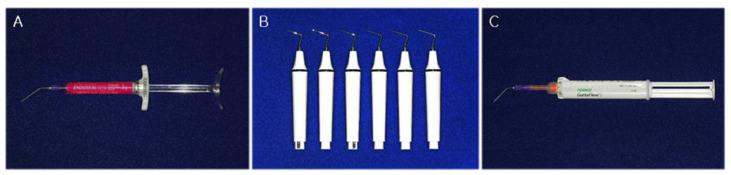
Materials used in this study. (**A**) Endoseal TCS calcium silicate-based root canal sealer and a blunt-end 24-G needle attached. (**B**) The VibraTHO system ultrasonic tips attached to ultrasonic handpieces (prototype). (**C**) GuttaFlow 2 root canal sealer with an intra-oral automixing tip and a blunt-end 24-G needle attached. VibraTHO: ultrasonic vibration and thermo-hydrodynamic obturation.

**Figure 2:**
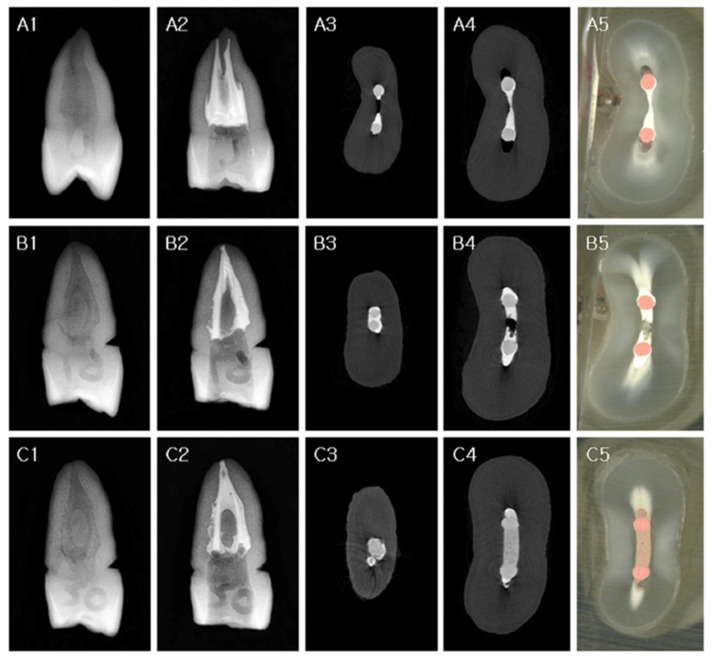
Representative images of each group from standard radiographs, micro-CT scans, and light microscopes. (**A1**–**A5**) SE group, (**B1**–**B5**) VE group, (**C1**–**C5**) VG group. Representative pre-operative standard radiographic images of each group (A1, B1, and C1). The specimens were filled using GP as follows: (A2) single-cone technique + Endoseal TCS, (B2) VibraTHO technique + Endoseal TCS, (C2) VibraTHO technique + GuttaFlow 2. Micro-CT scans of the cross-section of root canals at the apical (A3, B3, and C3) and middle thirds (A4, B4, and C4). Microscopic images of the sectioned root surface at the middle thirds (A5, B5, and C5), where the micro-CT cross-sectional images (A4, B4, and C4) were obtained. CT: computed tomography, GP: gutta-percha, VibraTHO: ultrasonic vibration and thermo-hydrodynamic obturation.

The conventional SC technique was used with the Endoseal TCS sealer in the SE group. Endoseal TCS was dispensed directly into the canals, no deeper than the coronal half, from a premixed syringe via a disposable blunt 24-G canal tip (Figure 1A). The prefitted master GP points corresponding to X3 of the ProTaper Next system were inserted simultaneously to their full length without any pumping or twisting motion. The excess GP was removed at the canal orifice level with a hot plugger, and the remaining GP point was vertically packed using a cold hand plugger (Figure 2(A1,A2)).

The VibraTHO technique was used with the Endoseal TCS sealer in the VE group. One or two ultrasonic tips of the VibraTHO system (prototype) were selected and attached to ultrasonic handpieces (dmetec, Bucheon-si, Korea), depending on the size that fitted the prepared root canal orifices (Figure 1B). The tip that bound in the canal at a depth of approximately 4 mm from the orifice was assumed to “fit” the canal. After dispensation of Endoseal TCS and insertion of the prefitted master GP points, as in the SE group, the root canal filling was completed using the VibraTHO technique as described in the previous study by Cho et al. [16]. The excess GP was sheared off at the orifice with the ultrasonic tip in an activated state, followed by 2–3 s of indirect ultrasonic sealer activation and heating of the GP with the activated tip. This was followed by 2–3 mm of short-range vertical compaction of the heat-softened GP with the deactivated tip after 1 s of cooling; 5–10 s of sustained vertical pressure at this position; finishing of the remaining GP with the same tip by turning the ultrasonic power on and off (Figure 2(B1,B2)). Only one tip of the VibraTHO system (prototype) was used to complete the whole task per canal.

The VibraTHO technique was used with the GuttaFlow 2 sealer in the VG group. The canal filling procedure used in this group was similar to that of the VE group, except that the sealer used was GuttaFlow 2. A small amount of silicone-based endodontic sealer, GuttaFlow 2 (Coltene/Whaledent GmbH + Co. KG, Langenau, Germany), was placed directly into the canals no deeper than the coronal half. This was conducted with an intra-oral automixing tip (SEIL GLOBAL, Busan, Korea), which was attached with a blunt-ended 24-G needle (Figure 1C). Subsequently, prefitted master GP points were inserted without any pumping or twisting motion and the root canal filling was completed using the VibraTHO technique (Figure 2(C1,C2)).

The teeth were maintained at 100% humidity for 7 days at 37 °C to allow the sealer to set completely after root canal filling.

### 2.3. Micro-CT Evaluation

A high-resolution micro-CT device SkyScan 1173 (SkyScan, Bruker, Billerica, MA, USA) was used to scan the teeth after filling the canals. The micro-CT scanner had the following specifications: a pixel size of 7.10 µm; X-ray source voltage, 130 kV; beam current, 60 µA; aluminum filter thickness, 1.0 mm; rotation step, 0.3° per step; exposure time, 500 ms. NRecon software version 1.7.0.3 (Bruker microCT, Kontich, Belgium) was used for image reconstruction. The filled area located 1–4 mm from the root apex was assigned as root canal area “A” (apical 3 mm) (Figure 2(A3–C3)). The rest of the filling was designated as the “MC” root canal area (middle-to-coronal) (Figure 2(A4–C4)) due to discrepancies in the root length among the samples. The sum of the “A” and “MC” areas was designated as “T” (total). The most apical 1 mm was not included in the analysis. The CT analyzer software (SkyScan) was used to measure the volume of the filling material and prepared root canal, and the volumetric percentage of filling material (V%) was calculated as follows:

V% = Vm/(Vm + Vv) × 100(1)

The volume of the prepared root canal is the sum of Vm and Vv, where Vm is the volume of the filling material, and Vv is the volume of voids. They were classified by grayscale. A grayscale ranging between 40 and 255 was assigned as the volume of the filling material (Vm), and a grayscale ranging between 0 and 40 was assigned as the volume of voids (Vv).

After micro-CT analysis, one tooth from each group was randomly selected and sectioned perpendicular to the longitudinal axis of the root using a low-speed diamond-coated saw (Isomet, Buehler, Chicago, IL, USA) under water cooling. The cross-sections were observed and photographed using a light microscope for verifying and validating the micro-CT evaluation method (Figure 2(A5–C5)).

### 2.4. Statistical Analysis

Statistical analyses were performed using SAS version 9.3 (SAS Institute Inc., Cary, NC, USA). The Shapiro–Wilk test was used to determine the normality of data distribution, followed by the analysis of variance to analyze the statistical differences between the three groups (*p* < 0.05).

## 3. Results

**Figure 3:**
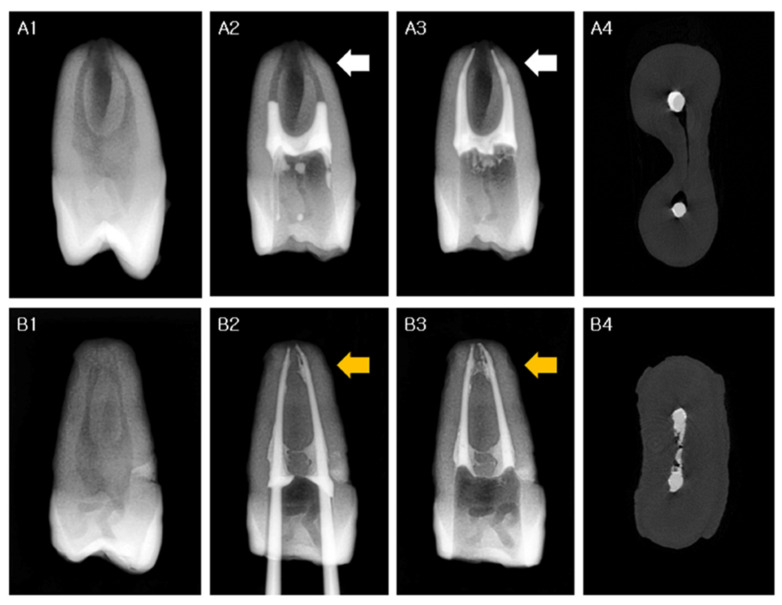
Representative images of root canal sealer movement at the apical thirds of SE group (**A1**–**A4**) and VG group (**B1**–**B4**). (**A1**,**B1**): pre-operative standard radiographic images of each group. (**A2**–**A4**): before (**A2**) and after (**A3**) single-cone technique + Endoseal TCS; micro-CT cross-section image at the apical thirds (**A4**), indicated by the white arrow in (**A2**,**A3**). Movement of Endoseal TCS into the complex apical anatomy can barely be noticed. (**B2**–**B4**): before (**B2**) and after (**B3**) the VibraTHO technique + GuttaFlow 2; micro-CT cross-section image at the apical thirds (**B4**), indicated by the yellow arrow in (**B2**,**B3**). Even though it is not perfectly filled, hydrodynamic movement of GuttaFlow 2 into the complex apical anatomy after application of the VibraTHO technique is clearly observed. CT: computed tomography, VibraTHO: ultrasonic vibration and thermo-hydrodynamic obturation.

The volumetric percentages of the filling materials were calculated, and the results were compared between each group at two canal levels and for the total area. The mean volumes (%) of the filling materials are shown in Table 2. The canal space was not obturated completely in any group (Figure 3). The mean volume of the filling material was higher in the VG (93.7–94.6%) and VE groups (88.9–91.6%) than in the SE group (86.5–70.6%) (*p* < 0.05) along the apical to middle-to-coronal thirds, and significant differences were observed between the groups in each root canal area (*p* < 0.05), with the only exception being at the apical thirds between the VE and SE groups (Table 2). Photomicrographs of representative cross-sections are shown in Figure 2; these were in good accordance with the micro-CT images taken at the same root canal levels.

## 4. Discussion

Studies on the quality of root fillings in canals in single-rooted teeth with simple configurations have reported remarkable results, even with the noncompaction SC canal filling technique [19,20]. However, cleaning and thorough filling of the root canal system become difficult if the root anatomy is complex [4,5,6]. This presents a dilemma for clinicians, since it is impracticable to precisely evaluate the complexity of root canal systems and the completeness of the root fillings clinically (Figure 3). However, only a few studies have investigated the root filling quality using the SC technique in complex root canal systems [10,11,17,18,21]. Considering these consequential issues, the present study was confined to human teeth with two root canals with complex anatomies, and the outcome of the noncompaction SC canal filling group with calcium silicate-based root canal sealer was quite divergent from that reported in previous SC filling studies using the same materials [8,10,11].

An analysis of studies on noncompaction SC obturation techniques with favorable results revealed that the apical portion of the specimens exhibited a comparatively higher percentage of root filling than the coronal portion in most cases [8,10,17,18,19,20,22]. The same result was verified in this study (Table 2). This finding should be interpreted with caution because some of the previous studies were conducted with canals of relatively simple anatomy, with a round configuration at the apical third; this feature can be observed in the representative figures of the specimens [19,20,22]. The lower filling scores for the coronal third can be explained by the discordance between the “round” GP points and the “oval” canals, since the SC technique does not entail any compaction effort to generate hydraulic pressure to compensate for this discrepancy. If the apical portion of the root canal system is also not round (akin to the coronal portion), which is not clearly discernible in clinical practice, apical canal filling with this method can result in less than ideal outcomes (Figure 2(A3) and Figure 3(A4)). The results of the VibraTHO technique using GuttaFlow 2 seem encouraging, apropos of this conundrum. The filling quality was significantly superior to that with the conventional SC method not only in the middle-to-coronal portions, but also in the apical portions with complex anatomy (Table 2, Figure 2(C3–C5) and Figure 3(B2–B4)).

The sealer placement methods used in studies focusing on the quality of SC obturation should be investigated with skepticism because they can influence the overall results [23,24,25]. Traditional obturation with compaction techniques is performed with a small amount of endodontic sealer that is delivered into the canal by coating the master GP cone or K-file with the sealer paste [3,4,14]. In contrast, the new premixed ready-to-use calcium silicate paste endodontic sealers are supplied in syringes and are intended to be injected directly into the root canals via needle tips [8,10,18,19,20,25]. This type of “syringe and tip” sealer placement method has been proven to effectively deliver the sealer evenly into the root canals [23] and significantly affect the total volume of obturation [25]. Moreover, a relatively large amount of root canal sealer can be delivered directly into the root canals by placing the dispenser tips deeper in the apical direction [8,11,14,26]. However, several SC canal filling studies have failed to explain the details of sealer placement protocols [10,19,20] or differentiated between methods used in groups that were created for comparison (i.e., the sealer-coated master GP point method for the compaction root filling group versus the “syringe and tip” sealer placement method for the noncompaction SC group) [8,11,14,25]. This can introduce bias into the comparison results [27].

Along with the increased depth of sealer injection into the canal with the “syringe and tip” method, another uncontrolled technical factor that is frequently found in studies on noncompaction SC obturation techniques is additional “pumping or twisting” action after the master GP cone insertion [8,13,24]. These factors can contribute favorably to sealer distribution for SC canal filling because the SC method does not entail any further compaction step to facilitate packing of the GP or sealer within the root canal. These factors may not be consequential in round canals with a simple anatomy [28], but can play a major role in the evaluation of the filling quality of root canals with complex anatomies (such as those observed in this study sample). Moreover, deep sealer injection or placement of the master GP cone with a pumping motion can also cause sealer extrusion from the apical foramen, which is clinically relevant [8,13,27]. Recent clinical studies have indicated that injectable premixed ready-to-use calcium silicate paste endodontic sealers tend to extrude beyond the apex [9]. Overextrusion of root sealers should not be considered completely harmless because it can cause serious irreversible complications in clinical practice [29,30].

Therefore, we controlled the sealer placement method and used the same protocol in each group to ensure the clinical validity of this study. Sealers were injected into the canals via 24-G needle tips, filling approximately half of the canals (Figure 3(A2)). The needle tips never extended beyond more than half of the canal length from the orifice. Master GP points were introduced very slowly to their working length, and further pumping motions were not used. Thus, the overall quality of the obturation in the noncompaction SC filling group was significantly poorer than that of the VibraTHO compaction groups (Table 2). This outcome raises grave questions about previous SC filling studies with uncontrolled sealer placement methods [8,10,11].

The long-term sealing of the root canal is influenced by the sealer [31]. The VibraTHO technique encompasses indirect ultrasonic activation of the root canal sealer, heat generation by ultrasonic energy to soften the gutta-percha, and geometrically driven short warm vertical compaction of the softened gutta-percha, which gives rise to the hydrodynamic stream of the sealer [16]. Although the results of the use of a calcium silicate sealer with the VibraTHO technique appeared to improve significantly at the middle-to-coronal portion compared to the SC technique, no significant improvement was observed in the apical area (Table 2). Moreover, the difference between the Endoseal TCS and GuttaFlow 2 groups with the VibraTHO technique was significant at the apical portion (Table 2). The GuttaFlow series is well known for its thixotropic properties [32] and excellent response to compaction canal filling procedures [15,26]. Notably, the movement of the GuttaFlow 2 sealer at the apical one-third was clearly verified in response to the VibraTHO technique in most specimens in the VG group (Figure 3(B2–B4)); however, this was not the case with the Endoseal TCS calcium silicate sealer in the VE group. Even though some laboratory studies reported the excellent thixotropic properties of calcium silicate-based endodontic sealers [12], a recent ex vivo study, which was conducted in the root canals of extracted teeth, reported a similar negative tendency as that observed in the present study [15]. Further studies with more clinically oriented criteria are needed to determine the detailed physical characteristics of calcium silicate-based sealers within the root canal.

Using extracted human teeth as specimens caused some limitations in this study. Because of the relatively small size of samples and limited groups of the experiment, we could not compare the individual effects of the physical properties of the root canal sealers in depth. Even with the overall group results, we cannot rule out the possibility that the difference in the results between the VG and SE groups stems from differences in the properties of the materials, not from differences in the techniques. Furthermore, we selected Endoseal TCS as the only calcium silicate-based root canal sealer for this investigation, but there are currently various products of this kind with different physical properties [33]. Finally, some uncontrolled experimental elements, such as root canal configuration, length and volume, and the amount of root canal sealer, cannot be overlooked. More controlled experiments with a standardized root canal configuration and amount of root canal sealer, and more groups of specimens with diverse combinations of canal filling techniques and root canal sealers are needed in the future.

## 5. Conclusions

Based on the results of this study, we conclude that the VibraTHO technique using GuttaFlow 2 can be a more effective root canal filling method for anatomically complex root canal systems than the SC technique with Endoseal TCS. On the other hand, the VibraTHO technique using Endoseal TCS has a limited effect on improving the quality of the root filling at the apical portion of anatomically complex root canal systems, compared to the SC technique with Endoseal TCS.

## Figures and Tables

**Table 1:** The canal filling techniques and root canal sealers assigned to each experimental group.

Group	Canal Filling Technique	Root Canal Sealer
SE group	conventional SC technique	Endoseal TCS sealer
VE group	VibraTHO technique	Endoseal TCS sealer
VG group	VibraTHO technique	GuttaFlow 2 sealer

SC: single-cone, VibraTHO: ultrasonic vibration and thermo-hydrodynamic obturation.

**Table 2:** Mean and standard deviation (SD) values of the percentage volume of gutta-percha + sealer at different canal levels in the 3 experimental groups.

Root Canal Area	Total (N = 30)	SE Group (n = 10)	VE Group (n = 10)	VG Group (n = 10)	Overall *p*-Value	Post-Hoc *p*-Value		
	Mean ± SD	Mean ± SD	Mean ± SD	Mean ± SD		SE vs. VE	SE vs. VG	VE vs. VG
A	89.7 ± 4.9	86.5 ± 4.7	88.9 ± 4.0	93.7 ± 2.8	0.0013 *	0.1699	0.0004 *	0.0125 *
MC	85.6 ± 12.9	70.6 ± 11.7	91.6 ± 4.0	94.6 ± 1.7	<0.0001 *	<0.0001 *	<0.0001 *	0.3492
T	88.6 ± 6.9	81.8 ± 6.9	90.0 ± 3.9	94.1 ± 2.1	<0.0001 *	0.0007 *	<0.0001 *	0.061

A: root canal area 1–4 mm from the root apex (apical 3 mm); MC: the remainder of the filled root canal area excluding A (middle-to-coronal); T: total of the filled root canal area (A + MC). SE group: single-cone technique + Endoseal TCS; VE group: VibraTHO technique + Endoseal TCS; VG group: VibraTHO technique + GuttaFlow 2. * Represents a significant difference between the groups at each canal level. VibraTHO: ultrasonic vibration and thermo-hydrodynamic obturation.

## Data Availability

The data presented in this study are available on request from the corresponding author.
